# CAR T-Cell therapy for the management of mantle cell lymphoma

**DOI:** 10.1186/s12943-023-01755-5

**Published:** 2023-03-31

**Authors:** Zoufang Huang, Vivek P. Chavda, Rajashri Bezbaruah, Hemant Dhamne, Dong-Hua Yang, Hong-Bing Zhao

**Affiliations:** 1 Department of Hematology, Ganzhou Key Laboratory of Hematology, The First Affiliated Hospital of Gannan Medical University, Ganzhou, China; 2 Department of Pharmaceutics and Pharmaceutical Technology, L M College of Pharmacy, Ahmedabad, , 380009 Gujarat India; 3 Department of Pharmaceutical Sciences, Faculty of Science and Engineering, Dibrugarh University, Dibrugarh, India; 4 Process Development, Gene Therapy Vector Facility, Research Management and Innvotations Directorate, King’s College London, London, WC2R 2LS UK; 5 New York College of Traditional Chinese Medicine, Mineola, NY 11501 USA; 6 Department of Oncology, the First Affiliated Hospital of Xinxiang Medical University, Xinxiang, China

**Keywords:** Mantle cell lymphoma, ZUMA, Brexucabtagene autoleucel, Chimeric antigen receptor T-cell therapy

## Abstract

Mantle cell lymphoma (MCL) is a subtype of Non-Hodgkin lymphoma (NHL) of mature B-cells characterized by translocation, which is typically due to excess expression of Cyclin D1. Although with the progress in our knowledge of the causes for MCL and available treatments for MCL, this cancer is still incurable. Age, male gender, rapid advancement, significant nodal involvement, elevated serum lactate dehydrogenase level, and prognostic indications including increased expression of Ki-67 and presence of TP53 mutation, are symbols of poor outcome. Advanced immunotherapy using chimeric antigen receptor (CAR)-T cells is advantageous for patients suffering from B-cell malignancies and MCL. Targeting B-cell antigens on the cell surface is a feasible approach in re-occurring (R/R) MCL because of significant responses obtained in other B-cell cancers. USFDA has approved brexucabtagene autoleucel (Tecartus, KTE-X19), a novel CAR T-cell therapy to be used in patients with MCL who have not responded to previous treatments or have relapsed. The FDA approved this new treatment depending on the outcomes of the ZUMA-2 clinical trial. Serious adverse reactions, moderate anti-tumor activity, allergen withdrawal, antigen escape, limited tumor infiltration, and trafficking are major barriers to successful CAR T-cell therapy. This review is a brief synopsis of the development of CAR T-cell therapy for MCL.

## Introduction

Mantle cell lymphoma (MCL) is a rare class of Non-Hodgkin lymphoma (NHL) and has a very varied course of manifestation. The terminology ‘mantle-cell lymphoma' was first coined by Raffeld and Jaffe in 1991 [1]. Mantle zone cells of primary lymphoid follicles are the source of this B-cell lymphoma. An outer circle of small lymphocytes encircling the germinal core of a lymphatic follicle is known as ‘mantle zone’ [2]. CD5-positive IgM-producing ‘B1a’ cells have been identified as the MCL's probable cell of origin [3]. This neoplasm expresses a distinctive immunophenotype, such as CD5^+^, CD10^+^, Bcl-2^+^, Bcl-6^+^, CD20^+^, and also IgM and IgD surface immunoglobulins, which provides mature B-cells a typical morphologic appearance [4, 5]. Most of the cases have the characteristic translocation of chromosome t (11;14) (q13: q32), which leads to excessive activity of the cell cycle regulator, cyclin D1 (CCND1/PRAD-1) gene [6]. CCND1 is a diagnostic feature for MCL because normal B lymphocytes do not express it [7, 8]. Although the cell cycle is dysregulated because of this oncogene's excessive production, it is not the only contributing factor. Instead, the progress of MCL is dependent on secondary oncogenic processes, like mutations that reduce DNA damage response systems [9].

CDKN2A deletions, CCND1 gene alterations, CDK4 amplification, TP53 mutations, SOX-11 overexpression, etc. are a few of the key pathogenetic aberrations of MCL [10]. The growth and progression of MCL depend on several variables, including SOX-11 cell cycle disruption, genetic changes, distinctive gene expression, epigenetic abnormalities, and microenvironmental milieu [11–13].

The symptoms of MCL are the same as that of NHL [14]. MCLs typically exhibit disease-related symptoms, such as lymphadenopathy, lymphocytosis, splenic, Waldeyer's ring and tonsil growth, lymphomatous polyposis, and bone marrow involvement [11, 15]. Patients, however, may continue to be asymptomatic despite lymphocytosis [16]. Kidneys, soft tissues, skin, the central nervous system (CNS), and other bodily sites are affected extranodal. Patients with non-nodal leukemic MCL frequently appear with asymptomatic indolent or ‘smouldering’ MCL [17].

In Western countries, MCL accounts for around 7% of adult NHLs, with a 4–8 case prevalence per million people each year [18]. MCL incidence rises with age. In the United States, the median age for the initial appearance of MCL is 68 years, whereas it is younger in Asian countries. Additionally, among the patients, three-quarters are men. Moreover, Caucasians experience the condition more frequently than those of other races do. Initially, MCL could only be detected in a highly limited, smaller subset of individuals with early-stage, low-risk diseases. However, the majority of patients require treatment quickly after diagnosis [19].

Despite improvements in current knowledge of the etiology of MCL and therapeutic strategies, it is still incurable. The initial evaluation of a certain MCL patient might define their treatment options. Chemoimmunotherapies based on Rituximab (R), with or without auto-SCT, are the usual first-line therapy for young, fit, and healthy individuals [19, 20]. Newer medicines that are chemotherapy-free targeted treatments are being researched as a result of the negative effects of chemoimmunotherapy, which include chronic myelosuppression, infections, and secondary malignancies [21]. Lenalidomide [22], Venetoclax [23], and other novel medications like Bruton tyrosine kinase inhibitors (BTKi; viz. Zanubrutinib, Acalabrutinib, ibrutinib) are beneficial but their duration of action is short, and patients typically relapse [24, 25]. Old age, male gender, blastoid variation, rapid progression, substantial nodal participation, augmented serum lactate dehydrogenase (LDH) level, and predictive indicators including elevated expression score of Ki-67 and existence of mutated TP53 are all factors that are indicative of poor outcome [26–29]. Consequently, to tackle the MCL challenge, innovative targeted agents are required.

Chimeric antigen receptor (CAR)-T cell therapy is a novel idea to the detriment of tumors because it was developed using headway in adoptive T cells and gene therapy [30]. The treatment involves taking T-cells from an infected person and altering the cells genetically to identify and destroy lymphoma cells [31]. Newer immunotherapy using CAR T-cells is advantageous for patients suffering from malignancies associated with B-cells [32]. Targeting B-cell antigens on the cell surface is a worthwhile approach in relapse and refractory MCL (R/RMCL) due to the significant responses obtained in other B-cell cancers [33]. This review is a brief synopsis of the development of CAR T-cell therapy for MCL.

## Treatment approaches for MCL

**Fig. 1:**
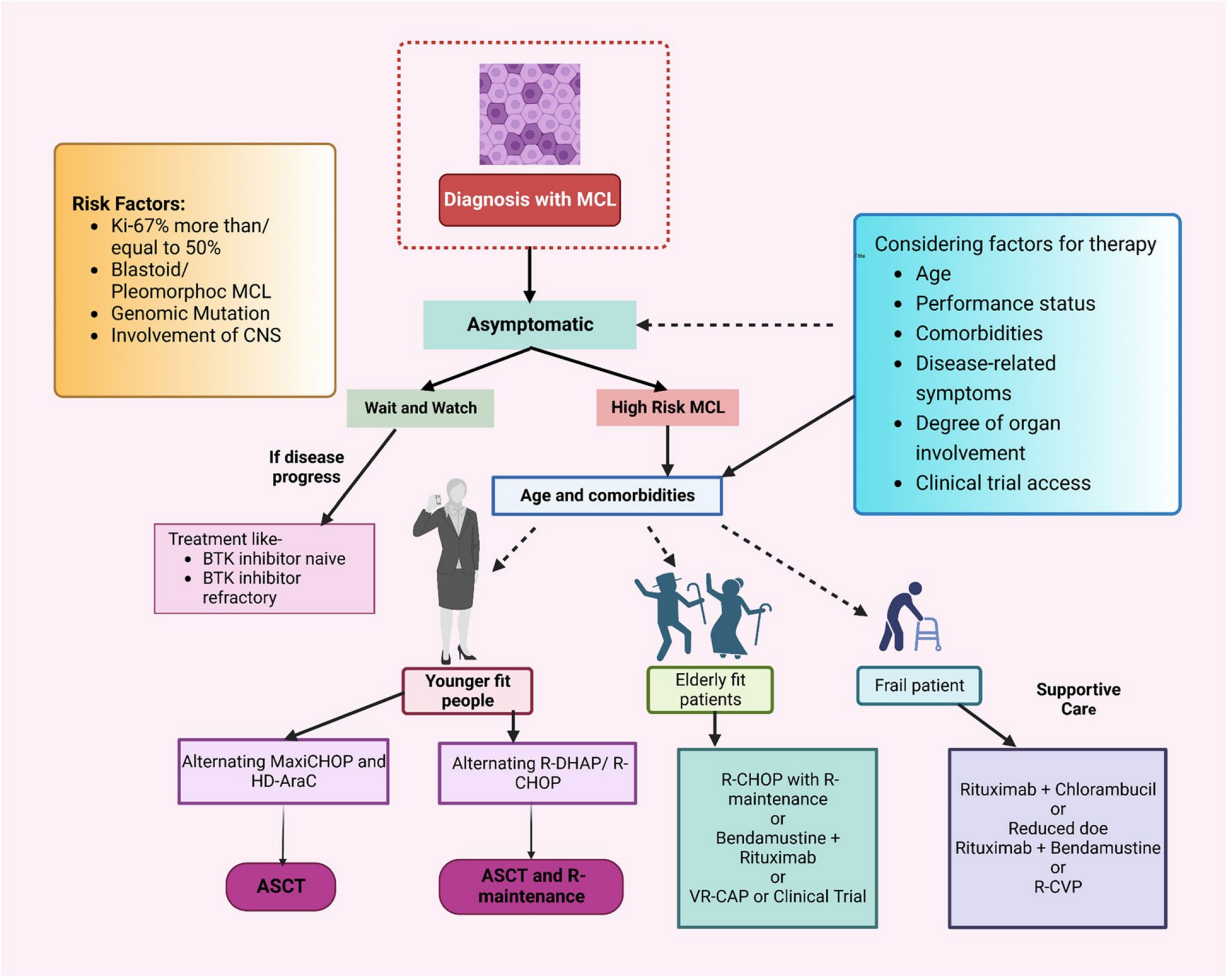
Existing approaches for first-line MCL therapy. When choosing a patient's course of treatment, it's crucial to take into account factors like age, performance level, the degree of clinical manifestations, eligibility for stem cell transplantation, multimorbidity, cardiovascular diseases, prognosis risk status, and convenience to clinical trials. High-risk individuals are often those who have blastoid/pleomorphic MCL, TP53-mutated or aberrant TP53, or Ki-67% > 30%. Abbreviations: ASCT, autologous stem cell transplantation; HD-AraC, high-dose cytarabine; R, rituximab; R-CVP, rituximab, cyclophosphamide, vincristine, prednisone; R-CHOP, rituximab, cyclophosphamide, doxorubicin, vincristine, prednisone; R-DHAP, Rituximab, dexamethasone, cytarabine, cisplatin; VR-CAP, bortezomib, rituximab, cyclophosphamide, doxorubicin, prednisone; CNS, central nervous system

The primary treatment strategy for MCL differs depending on age and associated diseases as summarized in Fig. 1. Young and physically active patients begin with intensive immunochemotherapeutic regimens containing Cytarabine, which are eventually combined with autologous stem cell transplantation (ASCT) [34]. However, for aged persons and patients with poor functional levels, chemotherapy of lesser intensity and continuous Rituximab treatment is the better alternatives; there are also nanotechnology-based interventions under development [1, 35–37].

One of these treatments is the Nordic regimen, signifies as maxi-CHOP, which contains high dose Vincristine, Doxorubicin, Cyclophosphamide, and Prednisone alternated with augmented Cytarabine dose and Rituximab [38, 39]. Other strategies are R-CHOP, which is alternated with Rituximab, Cytarabine, and Dexamethasone including a derivative of Platinum (R-DHAP). Rituximab and Bendamustine (RB) are given consecutively or alternately with high-dose Cytarabine (RC) [38, 40, 41]. Following autologous stem cell transplantation (ASCT), Rituximab administration has also been proven to improve overall survival [42].

It has been demonstrated that ASCT in the first remission enhances progression-free survival (PFS), but not overall survival (OS) [43]. As a result, the part of transplants is being re-evaluated in some studies. Rituximab after stem cell transplant is being studied in the ongoing ECOG-ACRIN 4151 randomized phase III trial to assess how well it performs in comparison to Rituximab alone in treating individuals who have been found to have ‘minimal residual disease-negative status' using next-generation sequencing (NGS) [44]. Although most young patients have favourable results, some high-risk categories don’t significantly benefited after intensive treatment, such as those with an elevated Ki-67 proliferation score or TP53 mutations [45].

Older individuals or those with associated diseases cannot sustain a high dose of Cytarabine, given randomized data contrasting them to R-CHOP, regimens including Rituximab and Bendamustine or Rituximab, Bortezomib, Prednisone, Cyclophosphamide, and Doxorubicin are favoured treatments [46–49]. Nevertheless, Rituximab and Lenalidomide may perhaps be advised for individuals who have not yet received therapy [50].

For patients with R/RMCL, chemoimmunotherapy plays a far smaller part in disease management than frontline therapy as relapses are frequent with poor prognosis [51, 52]. Targeted medications are typically used due to their safety and effectiveness. The first approved targeted therapies were Bortezomib, Temsirolimus, and Lenalidomide; nevertheless, Bruton Tyrosine Kinase (BTK) inhibitors have emerged as the key players in second-line treatment [28, 53, 54]. Ibrutinib, Acalabrutinib, and Aanubritinib are three BTK inhibitors that have so far been authorized by FDA for use in R/RMCL. Despite having short half-lives, these drugs are easy to dose because of how they covalently link to the BTK enzyme's Cysteine and cause irreversible inhibition [55]. In R/RMCL, combination treatment is also being extensively investigated. The combination of Ibrutinib with Venetoclax is very effective, producing results that are better than those of either drug used alone while maintaining a tolerable level of safety. Significant response rates were achieved as a result of the combined effect of inhibiting the BTK system and the BCL2 gene, yielding complete response (CR) of 42% (without a PET) and 62% (with a PET) at 16 weeks [56]. The effectiveness of the synergistic use of Ibrutinib and Venetoclax over Ibrutinib alone in R/RMCL is being analyzed in the clinical trial (Phase 3) SYMPATICO (NCT03112174) [28].

Effective therapy for patients who relapse following BTK inhibitor (BKTi) is an urgent clinical need. It is unclear what the best course of action should be for patients who improve after using a BTKi, however, Venetoclax, chemoimmunotherapy, and immunotherapy are options to consider for treatment of the disease. Retrospective analysis revealed that the Rituximab, Bendamustine, and Cytarabine (R-BAC) combination had a high objective response rate (ORR) (83%) in MCL survivors who had developed it following BTKi therapy. The R-BAC technique was used as a transitional treatment in transplant-eligible individuals before consolidating by ASCT, despite responses being not very long-lasting (PFS of 10.1 months) [57].

Furthermore, another class of drug, the BCL2 (B-cell lymphoma) inhibitors have the potential for treating MCL, where BCL2 is typically overexpressed. Venetoclax is a strong and a selective inhibitor of BCL2. Venetoclax had a 75% response rate (21 patients) and a 21% CR rate in a phase 1 study involving patients with R/RMCL [58]. Although BTK and BCL2 inhibitors have promising response rates, their usage may be constrained by the expansion of drug resistance. Evidence suggests that mutations in the BCL2 family of proteins are responsible for MCL's acquired resistance to Venetoclax [59, 60].

A potential trial evaluated allo-SCT as a rescue treatment for patients who had R/RMCL and as the first-line management of MCL, and both groups showed comparable outcomes with a 5-year overall survival of 73%. According to a trial, performed by Simon Rule et. al. an early allo-SCT could be advantageous for juvenile subjects with increased risk outlines, predominantly those who have mutations of TP53 [61].

Even if conventional treatment options have some limitations, the development of anti-CD19 CAR T-cell therapy has revolutionized the treatment of R/R lymphoid malignancies [62, 63]. CAR T-cell-based adoptive cellular immunotherapy offers effective and long-lasting therapeutic effects for a subset of patients with recurrent BCL2. According to the ZUMA-2 clinical trial research, it was discovered that CAR T-cell therapy is the most effective treatment for MCL and R/RMCL when compared to other treatment options [64].

## CAR T-cell therapy

**Fig. 2:**
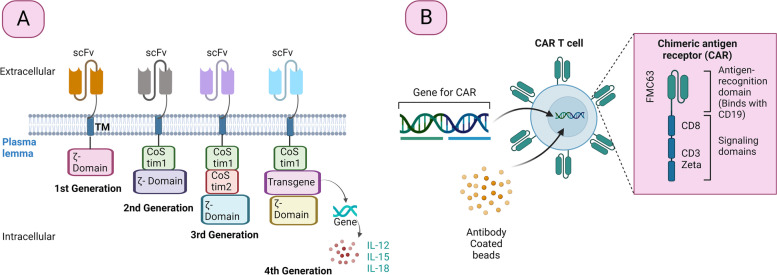
Different generations of CARs and manufacturing process of Tisagenlecleucel CAR. **A**. Generations of CAR. All the approved CARs are from the second generation. **B**. manufacturing process of Tisagenlecleucel CAR Abbreviations: scFv; variable fragments of a single-chain antibody, TM; transmembrane protein, CoStim; Costimulatory domain, IL; Interleukin

CAR is a modular protein, fused with 4 domains i.e., an extracellular domain of target binding generally obtained from variable fragments of a single-chain antibody (scFv), a transmembrane domain, a spacer domain, and CD3z (an intracellular domain of signalling linked with CD28, CD134, CD137 or many other co-stimulatory molecules depending on the CAR generation) [65]. It further results in independent cell activation through a major histocompatibility complex (MHC) [66]. From 1989 to 1993, two immunologists Gideon Gross and Zelig Eshhar from the Department of Chemical Immunology at the Weizmann Institute of Sciences, Israel, generated the first modified T-cell with the chimeric molecule [66, 67]. Figure 2A represents various generations of CARs. The CAR T cell’s first generation was perfectly adapted for stimulating cytolytic characteristics and giving selectivity like T-cells antibodies and causing damage to the targeted cell [68, 69]. Nevertheless, activating resting T-cells was not possible because the intracellular domain contains a single activation chain [70]. CAR-T cells need an additional co-stimulatory signal to operate effectively in the body, much like regular T-cell activity. Thus, to advance CAR-T cells' survival, multiplication, and antitumor activity, the second generation of CAR-T cells was shaped with an intracellular co-stimulatory domain [71]. The co-stimulatory molecules that are most frequently used are hematopoietic cell signal transducer (DAP10), CD28, Inducible T-cell costimulatory (ICOS), TNF receptor superfamily member 9 and 4 (4-1BB and OX40 respectively) [72].

In comparison to second-generation, the third-generation CARs had in vivo persistence and enhanced effector functions since it combines two co-stimulatory molecules like CD28 and 4-1BB (Fig. 2A) [73]. T-cells redirected for antigen-unrestricted cytokine-initiated killing (TRUCK), the fourth generation of CAR-T cells, have an additional transgenic expression cassette that upon activation allows the synthesis of cytokines. This promotes the death of targeted as well as bystander cells mediated by cytokines while activating the innate immune system [74].

The Memorial Sloan Kettering Cancer Centre team created the first operative CAR-T cells in 2002, when targeting a prostate cancer antigen. However, the first promising findings of CAR T-cell therapy were announced in 2011, which demonstrated a full reduction in individuals with chronic leukaemia [75]. Around the same time, significant advancements in the management of acute BCL patients including children, as well as adults were reported. Following these, on 30th August, 2017, the US FDA permitted Tisagenlecleucel (Kymriah) for the therapy of acute BCL in young adults and children [76].

In April 2015, Kite, a Gilead Company started a clinical trial (ZUMA-1) to check the effectiveness and safety profile of Axicabtagene ciloleucel (Yescarta) in adult sufferers having Refractory Aggressive NHL with a total of 307 participants [31]. The ZUMA-1 trial follow-up data for 2 years showed that Axicabtagene ciloleucel can evoke enduring responses, and an average of more than 2 years of life-span in patients with R/R BCL, along with a tolerable long-term safety profile [77]. On 18^th^ October 2017, the USFDA authorized Axicabtagene ciloleucel, as a treatment for R/R diffuses large B-cell lymphoma (DL-BCL) and other uncommon large B-cell lymphomas(LBCL), which is the first approved CAR T-cell therapy in history [78].

On June 28^th^, 2018 the European Medicines Agency (EMA) permitted CD19 CAR T-cell therapies like Tisagenlecleucel (Kymriah, Novartis) based on the ELIANA study and Axicabtagene ciloleucel (Yescarta, Gilead-kite) based on the ZUMA-1 study in R/R lymphoblastic leukaemia in adolescents and children and DL-BCL for adults [70].

The USFDA, on July 24, 2020, granted another novel CAR T-cell therapy Brexucabtagene autoleucel (Tecartus, KTE-X19), which is a second-generation CAR for MCL. The approval relied on the ZUMA-2 results in those MCL patients who did not show any response to earlier medications or who have relapsed [79, 80]

**Fig. 3:**
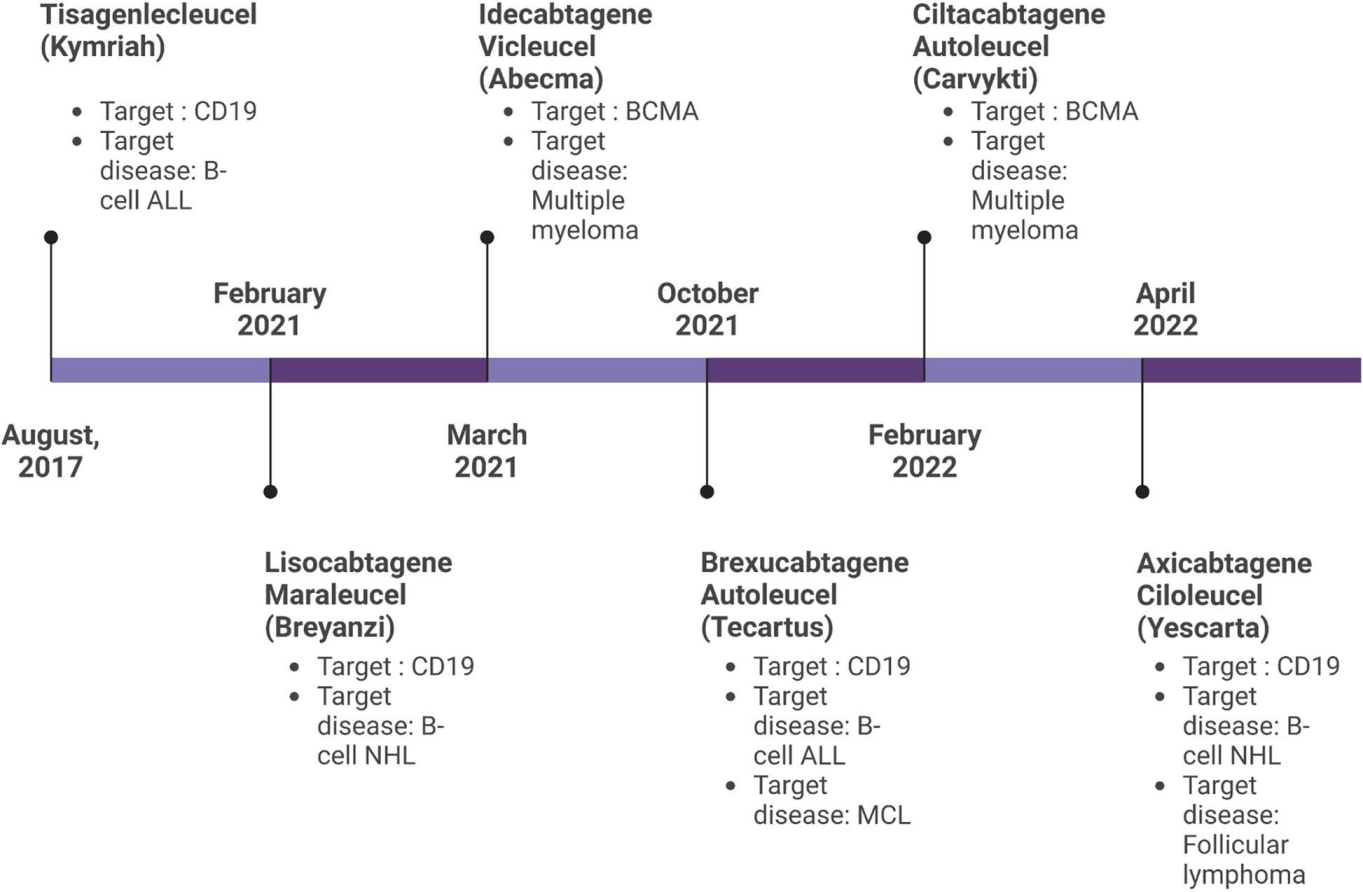
USFDA approved CAR T-Cell therapies

Since 2017, USFDA has approved six such therapies as a treatment for different blood cancers such as multiple myeloma and leukaemia. USFDA-authorized CAR T-cell therapies (Fig. 3) include Axicabtagene ciloleucel tisagenlecleucel, Idecabtagene vicleucel, Ciltacabtagene autoleucel, Lisocabtagene maraleucel, and Brexucabtagene autoleucel [81]. Among all of these treatments, Brexucabtagene autoleucel, which targets the CD19 antigen, is the most effective for MCL [82].

**Fig. 4:**
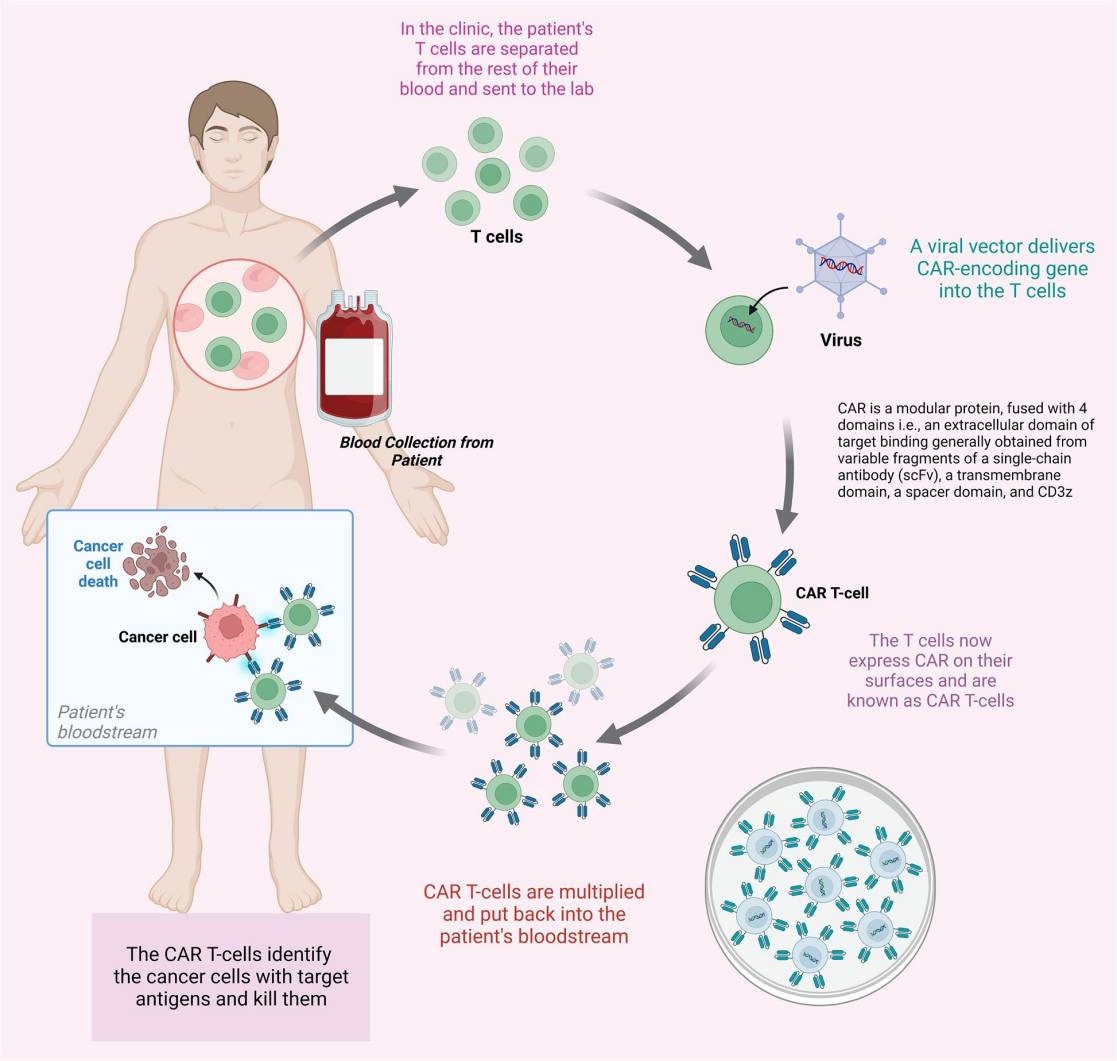
Process of CAR T-cell therapy. The therapy involves the modification of the T-cell of the patient. Here, the blood is collected through the vein and sent into the apheresis (Not present in the diagram) where it separates along with the white blood cell. The remanent of the blood is infused back into the patient. Gene for the special treatment known as CAR is administered with T-cell, which facilitates the binding. This complex is amplified in the laboratory and then reverted to the patient, which targets the cancerous mass and destroys them

The manufacturing process affects the effectiveness of CAR T-cell therapy. In contrast to other commonly used drugs, autologous CAR T-cells are created from the patient's T-cells, making this therapy safer and having more tolerable side effects [83]. T-cells are first removed from the patient's WBC during the manufacturing process. They are altered and activated to express CARs, giving them the capability to identify and eradicate tumor cells [32]. Following that, the modified T-cells are readministered to the patient [31]. The patient undergoes conditioning with lymphodepleting chemotherapy before re-administration [84]. The white blood cells are collected by conventional leukapheresis. Leukocytes are separated from the patient's blood, which is then eliminated from the body before the remaining blood is put back into circulation. Figure 2B and 4 depicts the manufacturing process of CAR T-cells.

After sufficient harvesting of leukocytes, the anticoagulant present in the leukapheresis buffer during the process is washed out from the leukapheresis product [85]. T-cells are concentrated by using the counter flow centrifugal elutriation process. This procedure also allows for the preservation of cell viability, the separation of leukocyte cells from other blood cells based on size and density, and for the enrichment of the leukapheresis product for lymphocytes [86]. The T cells are selectively enriched or isolated mainly using a magnetic bead-based technology CD3/CD28 beads or CD4 and CD8 beads. Then the cells are activated by culturing with a artificial antigen-presenting cells (APCs, primarily anti-CD3/CD28 beads) followed by transduction using a viral vector encoding for CAR [87]. The selection, activation, transduction, and expansion steps are performed aseptically in multiple systems such as G-Rex bottles, Gas Permeable Bags, CliniMACS Prodigy, WAVE/Rocking Motion bioreactor. The media contains a combination of cytokines such as IL2, IL7, IL15, or IL21 and human AB serum and the complete media is perfused or exchanged on alternate days to allow for rapid expansion [88]. The expansion takes anywhere from 6 to 21 days. During and at end of the process, the samples are taken out to determine the identity, safety, purity, and potency of the products. It includes expression of CAR receptor, T-cell purity, percent viability, cell number, sterility, absence of Mycoplasma, absence of replication-competent retrovirus, vector copy number, in vitro killing of B-cells and antigen-dependent cell proliferation and cytokine secretion, etc. [89].

### Usage of CAR T-cell therapy

#### Targeting cancer-associated fibroblasts (CAF)

CAFs, which narrates a crucial part in tumor initiation and development, are common stromal components in the tumor microenvironment (TME) [90]. CAR T-cell therapy aimed at Anti-Fibroblast Activated Protein (Anti-FAP) is the utmost promising immune-based therapy established to decrease CAF, but its success has been inconsistent. Several preclinical investigations on FAP-aimed CAR T-cells in conjugation with various anticancer medications for pancreatic, breast, and lung cancer have shown considerable anti-tumor activity [91]. Other trials using FAP-targeted CAR T-cells, reported less efficacy as well as fatal cachexia and bone toxicity as a result of off-target toxic effects triggered by the abolition of FAP + progenitor stromal cells [92]. Two clinical investigations have now confirmed the existence of anti-FAP CAR T-cells. Four patients with malignant pleural mesothelioma were engaged in a Phase 1 study employing FAP CAR T-cells in 2012 (NCT01722149). Another recent trial uses a fourth-generation CAR T-cell treatment that targets Nectin4/FAP to treat advancing malignant solid tumors (NCT03932565). Despite considerable progress in CAF biology, currently, the absence of precise markers of CAF cells, along with the variety of CAFs, hinders the application of CAR T-cell therapy to eradicate CAFs in the tumor microenvironment [91].

### Targeting tumor vasculature

In the current context, endothelial tumor cell depletion by CAR T- cells is a useful technique. CAR T-cell therapies targeting endothelial antigens have several advantages over traditional techniques, including genetic constancy and access to circulating T-cells [93]. This approach can also be employed in the management of solid tumors. Vascular endothelial growth factor receptor (VEGFR) is the most promising CAR T cell target endothelial candidate and multiple studies have shown its efficacy in a variety of preclinical scenarios [94, 95]. When CAR T-cells are used as monotherapy, however, their efficacy is generally limited due to the binding of CAR to circulating VEGF-A. Tumor endothelial marker 8 (TEM8), prostate-specific membrane antigen (PSMA), the NKG2D-ligand Rae1, αvβ3 integrin, and the EIIIB fibronectin splice variant are among the compounds being explored for CAR T-cell therapy aimed at vascular disruption [96]. In general, the off-target/on-target consequences of these CAR T-cell therapy candidates are still poorly considered [91].

### CAR T-Cell Therapy targeting B- cell Lymphoma

CAR T-cell therapy is the newer treatment that is found to be effective in R/R MCL patients. Four anti-CD19 CAR T-cell therapies (Kymriah™, Tecartus™, Yescarta™, and Breyzani™) are presently authorized for the treatment of BCL, follicular lymphomas, and MCL [97]. The anticancer activity of the CAR depends on vital components of CAR. In the instance of BCL, the single-chain variable component preferentially targets B-lineage cells irrespective of the MHC when it fixes to the antigen epitopes on a tumor cell.

By eliminating circulating CD19 expressing malignant cells, anti-CD19 CAR T-cell therapy, Axicabtagene ciloleucel (Axi-cel, KTE-X19, Tecartus™) is prepared for patients associated with MCL [98]. Axi-cel is an autologous cell therapy, like other CAR-T cell products, which conveys a protein complex containing a T-cell signalling domain (intracellular) and an antigen recognition domain (extracellular) (NCT02601313). An anti-CD19 antibody's changeable single-chain fraction known as the extracellular antigen-recognizing domain attracts changed T-cells to the surface of both healthy and cancerous B cells [99]. The additive domains CD28 and CD3-zeta enable subsequent signalling pathways when anti-CD19 CAR T-cells interact with target cells that express CD19. This causes T-cell stimulation, proliferation, the development of effector abilities, and the release of inflammatory chemokines and cytokines [63]. Because of the destruction of CD19-expressing cells, the exhaustion and activation of anti-CD19 CAR T-cells decrease during the ex vivo process of manufacturing [64]. Axi-cel was approved by the FDA for R/R large BCL in 2017 and shortly after by the European Medical Agency (EMA) in 2018. These approvals were based on optimistic phase-2 results from the ZUMA-1 study that reveal long-term remission and CR rates. Axi-111 cel's patients with refractory LBCL in a phase 2 trial demonstrated an 82% overall response rate and a 54% total response rate. Despite the fact that three patients died while undergoing treatment, the study's risk–benefit analysis revealed that CAR T-cell therapy showed appreciable levels of sustained response, with a safety profile that included myelosuppression, neurologic complications, and cytokine release syndrome [31].

Following this, Tisa-cel (Tisagenlecleucel; CTL019), was also licensed in 2017 for the treatment of R/R DLBCL according to the findings of the global, phase-2, pivotal JULIET study (NCT02445248). A total of 93 individuals received tisa-cel as part of the trial and assessed its effectiveness and safety. In total, 86% of patients showed grade 3 or 4 adverse events (AEs). Three individuals passed away due to disease progression 30 days after receiving the treatment. However, tisa-cel was not connected to any fatalities [100].

Another trial namely TRANSCEND examined the anti-CD19 CAR drug Liso-cel (Lisocabtagene maraleucel; JCAR017) is incorporated in the management of R/R LBCL (NCT03483103). Liso-cel displayed long-lasting clinical efficacy with a favourable safety profile. Among the 255 subjects, the ORR and CR rate were found to be 73% and 53% respectively; also, DOR was 55% and PFS was 44%, respectively, at 12 months. All histologic groups and patients with poor prognoses, such as those who were refractory, elderly, comorbid, and/or had a large tumor load, showed clinically significant effectiveness [101].

### CAR T-Cell Therapy for MCL

In MCL, there is less success using CAR T-cell therapy; however, two trials ZUMA 2 and TRANSCEND NHL 001 in patients with relapsed and resistant MCL have produced fascinating findings recently. Table 1 summarizes various clinical development outcomes for MCL. ZUMA 2 is a Phase 2, multicenter clinical study that aimed to look at the effectiveness of KTE-X19 in people who have R/R MCL (NCT02601313). A CD19-directed CAR T-cell therapy with a co-stimulatory domain of CD28, is used in ZUMA-2 CAR T-cell therapy. The trial started on November 9, 2015, by Kite, a Gilead Company. In phase 2 of the experiment, every brexu-cel goal dose contained 2 folds 106 viable CAR-positive -T cells/kg. Brexu-cel is a 68-mL infusion bag containing a frozen solution of autologous T-cells (genetically modified) in 5% dimethyl sulfoxide (DMSO) with 1% human serum albumin [97]. With just a single infusion of the medication, the study had a remarkable response rate. Aside from the manufacturing process, brexucabtagene autoleucel is exactly similar to axicabtagene ciloleucel. During the ZUMA-2 clinical trial, no statistical significance was found in any specific adverse predictive subgroups. Brexucabtagene autoleucel treatment was found to be safe, and effective, and has a remarkable response rate in older age patients, patients with blastoid variant and high Ki-67 proliferation index, and in those with a high risk of MIPI scores and TP53 mutation [102].

**Table 1:** Clinical developmental summary of CAR T-cell therapies for MCL

Phase	Intervention	Number enrolled	Adverse event	Remarks	Progression-free survival (PFS)	Overall survival (OS)	NCT number
Approved	Brexucabtagene Autoleucel (KTE-X19) [105]	74	Out of 33 patients, 91% develops CRS, 55% grade 2 and 3% patient with grade 3 adverse event	Best response was observed in 12% of total patients 3% of patient die within 1 month of infusion Overall response rate – 83%	PFS- 77% and 51% for post infusion of 6 and 12 month, respectively	OS- 83% and 61% post 6 and 12 months	NCT04162756
	Lisocabtagene Maraleucel (JCAR017) [106, 107]	184	Common grade 3 adverse event was observed as neutropenia (80%), anemia (49%), thrombocytopenia (49%)	Serious adverse event was observed in 48% patients •••Median follow-up was done at 6.2 months	PFS- 47% (at 2-year interval)	OS- 59% (at 2-year interval)	NCT03575351
	CD19 CART-T (expressing IL-7 and CCL19) [108]	39	Grade 3 cytokine and higher grader (> 3) was observed I 12.8% and 10.3%, respectively	Fourth generation Therapy	PFS- 53.8% after 13 months	OS- Greater than 2 years	NCT04833504
3	Drug: BTK inhibitor + Fludarabine + Cyclophosphamide + CAR-T-CD19 Cells Drug: Fludarabine + Cyclophosphamide + CAR-T-CD19 Cells	24	The study is ongoing and hence the data is not available	The study is ongoing and hence the data is not available	The study is ongoing and hence the data is not available	he study is ongoing and hence the data is not available	NCT05020392
2	M19 CAR T-cells	68	Not noted yet	Not yet recruiting	PFS will be noted upto 24 weeks post-infusion once the study starts	OS will be noted upto 24 weeks post-infusion once the study starts	NCT05155215
	Brexucabtagene autoleucel	90	N/A	N/A	Not yet received. PFS will be noted upto 7 years post-infusion	Not yet received. OS will be noted upto 7 years post-infusion	NCT04880434
	Biological: brexucabtagene autoleucel Drug: Axicabtagene Ciloleucel [109]	105	Toxicities were rare, only 3% of patient develops adverse event on the longer duration	Duration of action was noted as 28.2 months	PFS- 25.8 months	OS- 46.6 months	NCT02601313
	Anti-CD19/20-CAR vector-transduced T-cells [110]	100	Cytokine release cytokine -50% Grade 1 or 2 and grade are 36% and 14%, respectively	One person died in the clinical trial No cases of encephalopathy	PFS- 12 month (64%)	Data not available	NCT03097770
	CAR T-Cell (CAR-20/19-T Cells) [111]	65	Not reported	Dose of 2.5 × 106 cells/kg (*n* = 16)	PSI was 866–1109 and polyfunctionality was 40–45%	Data not available	NCT04186520
	Autologous Anti-CD19CAR-4-1BB-CD3zeta-EGFRt-expressing T Lymphocytes (CD19-Specific Chimeric Antigen Receptor) [112]	204	The study is ongoing and hence the data is not available	The study is ongoing and hence the data is not available	The study is ongoing and hence the data is not available	The study is ongoing and hence the data is not available	NCT01865617
	CTX112 (Anti-CD19 CAR-T Cell Therapy) [113]	120	Not available	Not available	Not available	Not available	NCT05643742
	MB-106 (CD20-targeted CAR T-cell therapy) [114]	287	No CRS or ICANS ≥ Grade 3	FDA Grants Orphan Drug Designation to MB-106	Not available	An overall response rate (“ORR”) of 96% and complete response (“CR”) rate of 75% observed in a wide range of hematologic malignancies including follicular lymphoma (“FL”), CLL, diffuse large B-cell lymphoma (“DLBCL”)	NCT05360238
	JCAR017 (lisocabtagene maraleucel) [115]	385	Adverse event of grade 3 was observed on 79% during treatment and 5% post-infusion	CAR T was present in blood for upto 4 years	PFS- 6.8 months (average) (40.6%)	OS- 27.3 month (average) (50.5%)	NCT02631044
1	Anti-CD19/20-CAR vector-transduced T-cells [110]	100	Fatigue, night sweats, hypotension, injection site reaction, leukopenia, and anemia	Duration of action was 94% and 74% for 6 and 12 months	PFS- 76% and 59% for 6 and 12 months, respectively	The study is ongoing and hence the data is not available	NCT03097770
	ADI-001(CD20 gamma delta CAR-T) [116]	78	Adverse event was observed in 78% in grade ½	78% overall and complete response rate and sustained durability in patients	PFS data not available	The study is ongoing and hence the data is not available	NCT04735471

CAR T-cell treatment has the ability to manage MCL resistance when compared with other existing drugs, according to Michael Wang, who conducted the ZUMA-2 experiment. In ZUMA 2, KTE-X19 was evaluated in 60 MCL patients who had previously been provided with up to five therapies [103]. Previous treatments were required to include a monoclonal antibody (anti-CD20), chemotherapy based on Anthracycline- or Bendamustine, and BTKi (a protein having a noteworthy part in the progress and existence of certain types of cancer, inhibition with Ibrutinib (Imbruvica) or Acalabrutinib (Calquence) [104].

Brexu-cel was very active in the cohort (*n* = 60) employed for the initial efficacy assessment, with 93% of ORR and 67% of CR. Whereas, in an intention-to-treat evaluation, all 74 participants had an ORR of 85%, with 59% attaining a CR. Brexu-cel had quick therapeutic responses, with 1-month median times to respond and 3-month median times to CR.

The investigators presented the safety and efficacy results as of December 2019, with a follow-up of 17.5 months, at the 47^th^ Annual Meeting of the European Society of Blood and Marrow Transplantation (EBMT) (14th–17th March 2021). According to the median follow-up (17.5 months) report, twenty-nine patients (48 percent) remained in response, including 70% of those who had a CR [117]. With lengthier follow-up, brexu-cel responses have remained consistent. With 32.3 months of follow-up, 39 percent of the trial's first 28 patients were still responding. Brexu-cel's long-term durability is unknown, but thus far, its efficacy is encouraging in a group of high-risk BTK inhibitor-refractory patients [117].

The ZUMA-2 clinical trial achieved remarkable and durable remissions, according to Frederick Locke, MD, Moffitt Cancer Institute, Tampa, Florida. It is also important to note that there was no statistical significance for any specific subgroups with poor prognoses when Dr. Locke and colleagues examined the continuing response rate stratified by high-risk or other characteristics often linked to worse outcomes with MCL. The rates of long-term response were the same for patients aged 65 and above. The long-term response rates for patients with blastoid variations were comparable to those for other individuals. The percentages of long-term remission were the same in patients with a high Ki-67 proliferation index. The same held true for TP53-mutated individuals as well as those with moderate or high MIPI (MCL International Prognostic Index) scores. Dr. Locke added that even in high-risk patients, the KTE-X19 CAR T-cell treatment is showing outstanding long-term response rates [64]. Every patient who received KTE-X19 experienced at least one AE. An AE of grade 3 or higher was present in 99% of the participants. The most typical AE was hematologic toxicity. Two patients (3%) who had both received conditioning chemotherapy experienced grade-5 AEs; in the second patient, the bacteremia was connected to both the chemotherapy and the brexu-cel infusion [64].

AEs of special significance for all CAR T cell treatments include immunologic effector cell-associated neurotoxicity syndrome and cytokine release syndrome (CRS). Out of the 91% of patients with CRS overall, 15% had CRS grade 3 or above. To treat CRS, 59% of patients received tocilizumab, while 22% received glucocorticoids [118].

Brexu-cel selectively aims at CD19-positive cells, hence aplasia of B-cell is a predicted adverse effect, resulting hypogammaglobulinemia. Flow cytometry analysis revealed B-cell aplasia in patients who experienced an objective response and effective CAR T-cell growth during the initial evaluation [82, 119]. In contrast, during the course of the experiment, B-cell aplasia was absent in every patient who did not have a response. Intravenous immunoglobulin infusions are given to 32% of patients to treat hypogammaglobulinemia. However, with extended follow-ups, those who had continuing responses at 12 months demonstrated symptoms of B-cell recovery [64, 117].

Furthermore, in TRANSCEND research (lisocabtagene maraleucel (liso-cel; Breyanzi)) along with other subgroups of lymphoma, MCL patients were also enrolled. As of December 2020, 32 patients received liso-cel infusions, while 41 patients completed collection. Liso-cel was highly effective in patients who received it, with an ORR of 84% and a CR of 59%. The individuals with blastoid morphology showed 75% response rate, according to the researchers. Excellent safety was shown for liso-cel in this population. Hematologic abnormalities made up the bulk of adverse AEs of grade 3 and above. 34% of people experienced a grade 3 or higher hematologic toxic impact that persisted through day 29 following infusion [120].

The haplo-CAR T, where the T-cells are derived from matched healthy donors within the blood relation, like siblings or children, has been shown to be effective for patients with refractory MCL. Haplo-CAR T-cells could effectively proliferate in vivo and had clinically significant antitumor activity without serious side effects [121]. The patient achieved a partial remission, with minimal residual disease.

### Limitations of CAR T-Cell Therapy

CAR T-cell therapy is not completely free from side effects and like other medications; it is related to some adverse effects. Despite this approach being most known for its antitumor efficacy in B-cell hematological malignancies that have relapsed or are resistant to treatment, it is nevertheless linked to a significant relapse rate. Serious adverse reactions, moderate anti-tumor activity, allergen withdrawal, limited tumor infiltration, and limited trafficking are all pitfalls to successful CAR-T cell therapy [122]. Numerous factors contributed to the initial CAR T-cell therapy's failure. For instance, in some individuals, either after infusion or throughout the manufacturing process, the generated CAR T-cells did not grow adequately inside the patient's body. In other instances, CAR T-cells were improperly produced, or the patients had underlying illnesses. The lack of sufficient research to maximize the therapeutic usefulness of CAR T-cells is another factor in the failure or limits of the initial CAR T-cell treatment [123].

*Antigen escape* or the development of tumor resistance to single antigen-targeting CAR constructs is one of the prime constraints of CAR T-cell therapy. Many techniques now rely on targeting numerous antigens to lessen this restriction [124]. In order to simultaneously target several target tumor antigens, these use tandem CARs, which is a single CAR construct that comprises two scFvs, or dual CAR constructions. Clinical investigations using CD19 and CD20 or CD19 and CD22 have shown that each of these approaches has a chance of producing sustained permanent remission rates [125]. T cell and CAR T-cell metabolic reprogramming methods both improve TME responses, activity, and effector function, or they mitigate the detrimental consequences of certain TME-specific alterations conducted by the tumour cells on the invading T lymphocytes [126].

*Off-tumor/on-target recognition*, insertional oncogenesis, anaphylaxis, graft versus host disease, and recognition of off-target antigens are some common adverse effects connected to CAR T-cell therapy [127]. Thus, to ensure therapeutic efficacy and prevent "on-target off-tumor" toxicity, antigen selection is essential in CAR design. The targets for MCL mainly have CD19 and CD22, which is expressed by healthy normal B-cells as well. As a result, CAR T-cells targeting either of these antigens are able to eliminate normal B cells. In this context, B cell aplasia is regarded as a measure for determining the efficacy and durability of CD19 and CD22 CAR T-cell therapies, and their success rate [128]. This limitation can be overcome by designing TME specific CAR T-cell therapy like targeting hypoxia of TME [129]. This technique was devised to limit CAR expression to to those CAR T cells that reside in the hypoxic TME (rather than those that reside in the non-hypoxic milieu of non-malignant tissues). Targeting tumor-specific post-translational changes, such as solid tumor overexpressed truncated O-glycans like Tn (GalNAca1-O-Ser/Thr) and sialyl-Tn (STn) (NeuAca2-6-GalNAca1-O-Ser/Thr), maybe a way to get around this constraint [130–132].

Additionally, interconnections between the microenvironment of the tumor, host, and CAR T- cell give a remarkable influence on CAR T-cell function. In addition, emerging and executing these treatments demands a diverse workforce. The common adverse effects associated with T-cell therapy may be delayed, immediate, severe, or mild type or it may continue through the lifespan of the genetically modified T-cell [127]. The toxicity depends on the interaction of CAR T-cells with the specific protein in patients’ falls into one of two categories; the other is caused by the activation of CAR T-cells and the elevated production of a cytokine storm. Cytokine release storm (CRS), neurotoxicity, macrophage activation syndrome (MAS), and hemophagocytic lymphohistiocytosis are among the symptoms of the systemic cytokine toxicity of CAR T-cells (HLH)[133]. A toxic effect known as CRS is caused by the in vivo multiplication of CAR T-cells and triggers the release of several cytokines as well as a systemic inflammatory response. There are several grading scales for the severity of CRS, including variants of the Lee criteria, the American Society for Transplantation and Cellular Therapy (ASTCT), the Common Terminology Criteria for Adverse CAR T-Cell Therapy-Associated Toxicity (CARTOX), and the Penn criteria [134]. Immune effector cell-associated neurotoxicity syndrome (ICANS) is how the ASTCT classifies neurotoxicity in this circumstance. Lethargy, psychosis, encephalopathy, ataxia, convulsions, restlessness, and, in extremely rare circumstances, cerebral edema are only a few of the signs of neurotoxicity. ICANS scores are evaluated using a 10-point immune effector cell encephalopathy (ICE) grade, whereby evaluates mental health using a condensed set of inquiries concerning following instructions, identifying objects, focusing, and writing [135].

Although the FDA approved CAR T-cell therapy after stripping away some significant barriers, the drug seems to still have some side effects. For example; fever, hypotension, infections, encephalopathy, tiredness, tachycardia, and arrhythmia are all typical unwanted effects of brexucabtagene. FDA has issued warnings related to these side effects while continuing use of the therapy. Although the majority of side effects developed within the first two weeks of treatment, the FDA warned that some could appear later [136]. Due to these risks, brexucabtagene was authorized with a mitigation strategy and risk assessment to guarantee its safe use. A boxed warning for the potential of CRS and neurologic toxicities is included on the product label for brexucabtagene autoleucel [63].

According to Dr Wang, three of the trial's 60 subjects died due to treatment-associated side effects. In addition, deprived of CAR-T cell treatment, all of the affected people in the study might have died of MCL in less than a year. Dr Wang stated that more research was necessary to confirm the average length of time patients required for treatment [137]. This authorization of CAR T-cells seems to be another indication of personalized therapies using a patient's immune system to fight cancer, while also utilizing a scientific breakthrough in this new promising area of medicine. KTE-X19 is linked to a risk of cytokine release syndrome, a potentially fatal condition, as well as neurocognitive adverse reactions. KTE-X19 is only accessible through a Risk Evaluation and Mitigation Strategy (REMS) program due to these significant threats. This REMS program is identical to that of the same company's other CAR T-cell therapy (axicabtagene ciloleucel; Yescarta) [138].

Mark Roschewski, MD, a specialist in lymphoma at the NCI's Center for Cancer Research, stated of the therapy: "This therapy represents a tremendous development for the treatment of recurrent and treatment-resistant MCL and should be evaluated in all patients with the illness. According to Dr. Roschewski, the approval will be crucial for the treatment of individuals whose malignancies are resistant to BTK inhibitors. These patients have a poor prognosis overall, and some quickly progress to a more severe form of the illness after using BTK inhibitors [137].

Zanubrutinib, Ibrutinib, and Acalabrutinib, three authorized targeted BTK inhibitors, have altered the landscape of the management of R/R MCL. There have been recently released clinical trial (phase 1) findings for Tirabrutinib (ONO-4059/GS-4059) and another trial (phase 2) data for Orelabrutinib (ICP-022), two second-generation BTKi medicines that are currently being developed. These substances could soon be used as developing MCL therapy alternatives [139].

### Management of Toxicity and resistance of CAR T-cell therapy

Notwithstanding the above-mentioned successes, CAR T-cell therapy has a high probability of giving rise to several side effects, such as neurotoxicity, cytokine release syndrome, on-target/off-tumor recognition, insertional oncogenesis, graft vs host disease and anaphylaxis [123, 140]. Like Axicabtagene ciloleucel, Brexucabtagene autoleucel also has some adverse effects like cytokine release syndrome and neuronal toxicity, but these adverse effects can be easily managed and are self-limiting [141]**.** To overcome the systemic cytokine toxicities researchers have introduced different methods such as CAR subunit dimerizing agents, CAR T-cells associated with off/on switches on small molecule adapter agents, downstream signalling inhibitors of CAR, control of CAR protein expression by using protease inhibitors, and engineered modified CAR T-cells with the capability to synthesise factors (It can counteract the cytokine storm, and the capacity to control the CAR expression) [142].

### Management of neurotoxicity by rabbit Antithymocyte globulin (ATG)

Following CAR T-cell treatment in R/R MCL patient, cerebral edema can be deadly. However, a study evaluated that, cerebral edema because of CAR T-cell treatment can be completely recovered by multimodal clinical intervention that included rabbit antithymocyte globulin (ATG). After ATG injection, biomarker data demonstrate initial and vigorous CAR T-cell growth and associated production of inflammatory cytokines, then swift reductions in CAR T-cell and proinflammatory cytokine levels. This clinical data sheds information on the promising utility of ATG in the management of severe neurotoxicity caused by CAR T-cells [143].

### Management of toxicity by Engineered CAR T-cells

To be therapeutically beneficial, the CAR T-cell must stay inside its therapeutic window since toxicity results from exceeding the therapeutic window [144]. The quantity of tumour antigen produced by cancerous cells, tumour load, the affinity of the antigen-binding domain to its target epitope, and the costimulatory component of the CAR all influence the activation rate of CAR T-cells. In order to maximise therapeutic effectiveness and reduce toxicity, it is crucial to carefully analyse a number of CAR's modular components [145]. It would be expected that as the antigen-binding domain's affinity decreases, larger antigen densities on tumour cells will be needed in order to achieve significant levels of activation. Antigen-binding domains with micromolar affinity were substantially more selective for cancers with higher levels of target antigen expression than antigen-binding domains with low nanomolar/subnanomolar affinity [146]. By altering the hinge and transmembrane areas of activated CAR T-cells, it is also feasible to control cytokine production. Alteration of the CD8α-derived transmembrane amino acid sequences and hinge reduced cytokine release and CAR T-cell proliferation in a CD19-targeted CAR [147]. Another customizable area in CAR design is the costimulatory domain, for instance, by employing less developed subsets of T-cells or designing CAR T-cells with 4-1BB ligand, which creates a supportive environment for CAR-T cells, the *in-vivo* efficacy of CAR T-cells can be improved. By overcoming physical obstacles and antigen heterogeneity in solid tumors, CAR T-cell infiltration into solid tumors can be improved [133].

### Management of immunogenicity of CAR

The general toxicity of traditional chemotherapeutic drugs can be decreased by the ability of modified immune cells to recognize their target. Pharmacological immunosuppression, targeted activation, and expression of some elimination genes in CAR T-cells are some common steps to minimize the adverse effects of CAR T-cell therapy [127]. Using human or humanized antibody fragments instead of murine-derived CARs to reduce CAR immunogenicity may be helpful because the host immune system's detection of CAR constructs may contribute to cytokine-related toxicities [148].

#### Management of neurotoxicity by Modifying CAR transduced T-cells

Myeloid cells and cytokines appear to play a substantial role in CAR T-cell-induced neurotoxicity, since reports have demonstrated large increases in CD14 + cells in patients with grade 3 or greater neurotoxicity [149]. According to a pivotal large B-cell lymphoma CAR T-cell clinical trial, GMCSF increase was the blood biomarker most strongly linked to the onset of grade 3 or higher neurotoxicity [150]. Lenzilumab inhibits the macrophage and monocyte activating cytokine Granulocyte–macrophage colony-stimulating factor (GM-CSF) in preclinical trials, which results in a reduction in neurotoxicity and CRS and an increase in CAR T-cell activation [151, 152]. Similar outcomes seem to be produced by GM-CSF mutational inactivation in T cells that have been CAR-transduced. Thus, these results imply that GM-CSF neutralisation aids in lowering neurotoxicity and CRS [153]. Additionally, catecholamine and cytokine levels are reduced when tyrosine hydroxylase is deleted in a manner that is unique to myeloid cells or when this enzyme is inhibited by metyrosine. Furthermore, according to preclinical research, CD19-targeted CAR therapy for leukemia/lymphoma animal models reduced neuroinflammation when IL-1 receptor antagonists were used [154, 155].

### Suicide gene strategies of CAR

Suicide gene or "off-switches" techniques, through the use of a secondary inducing factor, would make it easier to selectively reduce engineered cells when unfavourable events start to occur [156]. For example, CAR designed to express full length CD20 or CD20 mimotopes help CAR-T cells be eliminated, through the use of rituximab [157]. The main drawback of these treatments, or those that are comparable to them, is that while they are desirable for assuring safety, their use abruptly ends treatment for a disease that is developing quickly. This restriction has provided a tremendous impetus for the development of safety-enhancing techniques that reserve suicide gene activation as a last option [158].

To increase results and get beyond CAR T-cell constraints, the right efforts must be made from the identification of patients here to separation, production, multiplication, and injection of CAR T-cells. To avoid CAR T-cell therapy restrictions or failure, it is crucial to monitor CAR T-cell effectiveness and antigen loss throughout treatment [123]. Improvement of conventional CAR-T cell design is necessary to overcome inadequacies or adverse effects of this therapy [133]. To assess the long-term protection of KTE-X19, the FDA has mandated that the manufacturing company initiate pharmaco-vigilance survey research of people who underwent KTE-X19 therapies. Due to the high cost and labour requirements, studies have begun to focus on allogeneic CAR T infusion, and these under different stages of clinical development. Transfusion of CAR T-cells is often a concern that hinders further progression and consequentially leads to cytotoxicity. However, CAR T-cell treatment modalities are still relatively new, and the widespread use of CAR T-cell therapy will undoubtedly confront a multitude of challenges in scientific knowledge before it can be extensively used [159].

## Conclusions

MCL is a pathologically unique and life-limiting disease that necessitates innovative therapeutic approaches. Providentially, the development of the CAR-T cell approach and cellular immunotherapy has opened up a novel world of therapeutic options. Researchers are investigating the CAR T-cells for different types of tumors, but until now, the most successful CAR T-cell therapy has involved only for the blood cancers. The majority of the CAR T-cell treatments carry the possibility of serious side effects, which must be handled quickly and carefully by medical professionals who are well versed in the procedure. In addition, Patients and healthcare providers experience distinct logistical and financial challenges. Severe toxicities like cytokine storm, macrophage activation syndrome, neurotoxicity, and HLH in patients due to the CAR T-cell therapy are some other unavoidable obstacles [160]. To minimize such events, researchers are working towards safety aspects of the CAR T-cell therapy by either increasing the selectivity of the CAR through safer choices of antigens, or alteration of the sensitivity of single-chain variable fragments of an antibody. It can also be possible by combinatorial antigen targeting and masking of the CAR, or by controlling the CAR T-cell activity by introducing possible suicide genes and limiting the CAR expression [161]. The real-world outcomes of brexu-cel for the treatment of R/R MCL in the United States were documented in a recent follow-up from July 2020 to December 2021. As per the report, ORR was 84% for all patients and 78% for patients with less than 180 days of follow-up. At six months, OS and PFS were 79% and 66%, respectively. After receiving tocilizumab and corticosteroids, grade 3 ≥ CRS (9%) and ICANS (29%) had mostly subsided by 21 days after initiation. 32% of patients acquired severe infections, 22% of patients had chronic cytopenia, and 3% of patients developed subsequent malignancies [162]. This is the most comprehensive report on the application of brexu-cel. This suggests the success of CAR T-cell therapy in MCL including those with high-risk features however, severe toxicities demands further investigation. Even though further research is necessary MCL therapy, there is hope that these trials would lead to a new medication in the therapy of lymphoma and would present a better cure for patients with an advanced type of cancer. These trials would pave a new way for research into different types of lymphoma and alter the lives of these previously difficult-to-treat individuals.

## Data Availability

Not applicable.

## Abbreviations

ASCT: Autologous stem cell transplantation
ATG: Antithymocyte globulin
BTKi: Bruton tyrosine kinase inhibitors
CCND1: Constitutive overexpression of Cyclin D1
CD: Cluster of differentiation
CAF: Cancer-associated fibroblasts
CR: Complete response
CAR: Chimeric antigen receptor
CNS: Central nervous system.
CRS: Cytokine release storm
DLBCL: Diffuse large B-cell lymphoma
FDA: Food and Drug Administration
HLH: Hemophagocytic lymphohistiocytosis
HD-AraC: High-dose cytarabine
ICANS: Immune effector cell-associated neurotoxicity syndrome
LDH: Lactate dehydrogenase
LBCLs: Large B-Cell lymphomas
MAS: Macrophage activation syndrome
MCL: Mantle cell lymphoma
MCL: Mantle cell lymphoma
NHL: Non-Hodgkin lymphoma
NGS: Next-generation sequencing
OS: Overall survival
ORR: Objective response rate
PFS: Progression-free survival
R/R: Relapsed and refractory
R: Rituximab
R-CVP: Rituximab, cyclophosphamide, vincristine, prednisone
R-CHOP: Rituximab, cyclophosphamide, doxorubicin, vincristine, prednisone
R-DHAP: Rituximab, dexamethasone, cytarabine, cisplatin;
RB: Rituximab and Bendamustine
RC: Rituximab and high-dose cytarabine
SCT: Stem cell transplantation
TEM8: Tumor endothelial marker 8
TME: Tumor microenvironment
VR-CAP: Bortezomib, rituximab, cyclophosphamide, doxorubicin, prednisone
VEGFR: Vascular endothelial growth factor receptor ()
